# Predicting arrhythmia recurrence following catheter ablation for ventricular tachycardia using late gadolinium enhancement magnetic resonance imaging: Implications of varying scar ranges

**DOI:** 10.1016/j.hrthm.2022.05.021

**Published:** 2022-05-26

**Authors:** Pranav Bhagirath, Fernando O. Campos, Caroline M. Costa, Arthur A.M. Wilde, Anton J. Prassl, Aurel Neic, Gernot Plank, Christopher A. Rinaldi, Marco J.W. Götte, Martin J. Bishop

**Affiliations:** * School of Biomedical Engineering and Imaging Sciences, King’s College London, London, United Kingdom; † Department of Cardiology, St. Thomas’ Hospital, London, United Kingdom; ‡ Department of Cardiology, Amsterdam University Medical Center, Amsterdam, the Netherlands; § Gottfried Schatz Research Center, Division of Biophysics, Medical University of Graz, Graz, Austria

**Keywords:** Arrhythmia recurrence, Cardiac magnetic resonance imaging, Catheter ablation, Late enhancement magnetic resonance imaging, Ventricular tachycardia

## Abstract

**Background:**

Thresholding-based analysis of late gadolinium enhancement cardiac magnetic resonance (LGE-CMR) can create scar maps and identify corridors that might provide a reentrant substrate for ventricular tachycardia (VT). Current recommendations use a full-width-at-half-maximum approach, effectively classifying areas with a pixel signal intensity (PSI) >40% as border zone (BZ) and >60% as core.

**Objective:**

The purpose of this study was to investigate the impact of 4 different threshold settings on scar and corridor quantification and to correlate this with postablation VT recurrence.

**Methods:**

Twenty-seven patients with ischemic cardiomyopathy who had undergone catheter ablation for VT were included for retrospective analysis. LGE-CMR images were analyzed using ADAS3D LV. Scar maps were created for 4 PSI thresholds (40–60, 35–65, 30–70, and 45–55), and the extent of variation in BZ and core, as well as the number and weight of conduction corridors, were quantified. Three-dimensional representations were reconstructed from exported segmentations and used to quantify the surface area between healthy myocardium and scar (BZ + core), and between BZ and core.

**Results:**

A wider PSI threshold was associated with an increase in BZ mass and decrease in scar (*P <*.001). No significant differences were observed for the total number of corridors and their mass with increasing PSI threshold. The best correlation in predicting arrhythmia recurrence was observed for PSI 45–55 (area under the curve 0.807; *P =* .001).

**Conclusion:**

Varying PSI has a significant impact on quantification of LGE-CMR parameters and may have incremental clinical value in predicting arrhythmia recurrence. Further prospective investigation is warranted to clarify the functional implications of these findings for LGE-CMR–guided ventricular ablation.

## Introduction

Catheter ablation for ventricular tachycardia (VT) has been proven to significantly reduce therapy and shocks from implantable cardioverter-defibrillators (ICDs).^1^ However, current guidelines for patients with an ischemic cardiomyopathy recommend catheter ablation only in case of recurrent ICD shocks due to sustained VT.^2^ There is limited evidence and therefore only a class IIa recommendation for patients with ischemic cardiomyopathy suffering from their first episode of VT. This is primarily due to the relatively low procedural efficacy and low long-term success rates of ablation therapy.^3^ An important explanation lies in the complexity and incomplete understanding of the arrhythmogenic substrate.^4^

Recent developments in cardiac magnetic resonance (CMR) postprocessing allow for detailed 3-dimensional (3D) scar characterization after myocardial infarction, including size (scar burden), transmurality (endocardium vs epicardial), and composition (scar core vs border zone [BZ]).^5^ Thresholding-based analysis of late gadolinium enhancement cardiac magnetic resonance (LGE-CMR) images can be used to create scar maps depicting viable corridors in areas with scar. This allows for noninvasive identification of electrical conduction pathways in damaged myocardial tissue (isthmus corridors), the anatomic substrate for arrhythmias. Scar maps, which contain information about corridors, can be merged with invasive electroanatomic maps to facilitate catheter ablation guidance.

Clinical scar quantification most often uses a full-width-at-half-maximum thresholding approach, effectively classifying areas with a pixel signal intensity (PSI) >40% as BZ and >60% as scar. This threshold is derived from a single study in a very limited number of patients.^6^ A cross-validation of this approach in a larger cohort is still lacking. Therefore, the impact of variations in these predefined thresholds on the generated scar maps and subsequent prediction of the arrhythmogenic sensitivity of the underlying myocardium remains unknown.

The aim of this study was to investigate the influence of 4 different threshold settings on scar quantification and sub-characterization in an ischemic cardiomyopathy patient cohort. In particular, the impact of threshold changes on identifying potential corridors and the association of all quantified scar metrics in predicting ventricular arrhythmia recurrence after catheter ablation was examined.

## Methods

### Patient population

A retrospective search was performed of the electronic patient records at the Amsterdam University Medical Centers to identify patients with an ischemic cardiomyopathy who had undergone catheter ablation for VT during a period of 4 years (2017–2021). Ethical committee study procedures were in accordance with the Declaration of Helsinki as revised in 2013. The study received approval from the institutional scientific board. Written informed consent was obtained from the study subjects. Patient data consisting of clinical characteristics, including follow-up and arrhythmia recurrence (therapies adjudicated by 1 physiologist and 1 device cardiologist), and CMR imaging data were downloaded and anonymized.

### Image acquisition

LGE-CMR images were acquired using a 2-dimensional (2D) inversion recovery gradient echo sequence in the short-axis orientation. Typical imaging parameters were spatial resolution 1.3 × 1.3 × 5 mm, TR 782 ms (repetition time = amount of time between successive pulse sequences applied to the same slice), TE 4.38 ms (echo time = time between delivery of the radiofrequency pulse and receipt of the echo signal), and flip angle 25° (amount of rotation experience by the net magnetization during application of a radio-frequency pulse). Imaging was performed 10–15 minutes after injection of 0.2 mmol/kg Dotarem (Guerbet Group, Villepente, France) contrast agent on a 1.5-T magnetic resonance imaging scanner (Avanto, Siemens Healthcare, Erlangen, Germany).

### Image postprocessing

**Figure 1:**
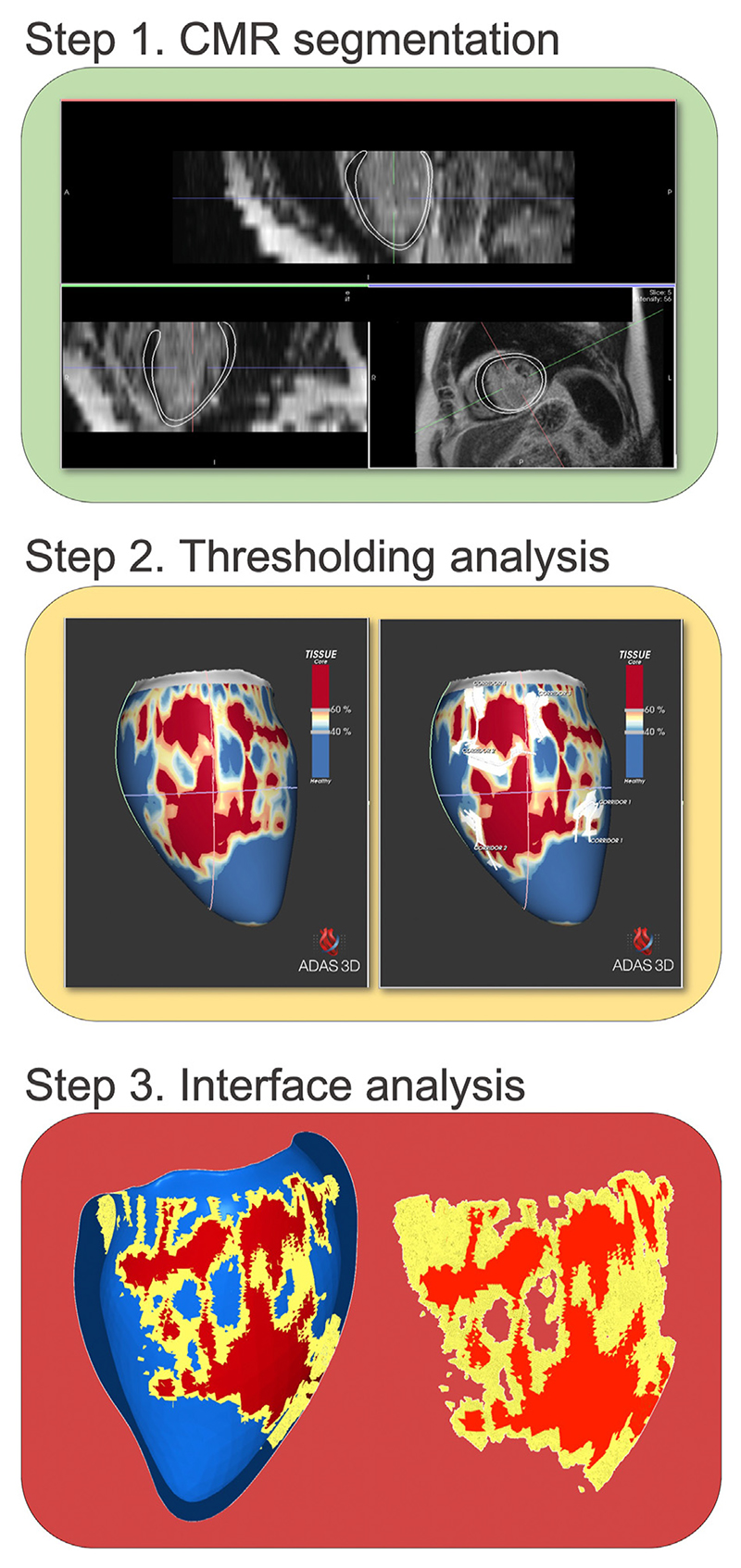
Image postprocessing workflow. Step 1 shows segmented *(white contours)* short-axis and two long-axis views of a 2-dimensional late gadolinium enhanced stack of the left ventricle. Step 2 depicts the thresholding analysis in ADAS with tissue characteristics on the **left** and corridor analysis on the **right**. Step 3 **left** shows a clipped view of the left ventricle mesh depicting healthy myocardium *(blue)*, border zone *(yellow)*, and scar core *(red)*. Step 3 **right** shows the extracted interface area of border zone and scar core. CMR = cardiac magnetic resonance.

Postprocessing was performed using ADAS3D LV (ADAS3D Medical, Barcelona, Spain),^7^ custom-written software, and an open-source meshing program (Meshtool).^8^ The work flow is shown in Figure 1. ADAS was used to semi-automatically segment the LGE-CMR images and create a left ventricular (LV) model. This model consisted of concentric layers from endocardium to epicardium and was used to create color-coded pixel signal intensity (PSI) maps of the LGE images with a full-width-at-half-maximum algorithm.

Four different threshold configurations were applied to subcharacterize the hyperenhanced myocardium on the PSI maps. The conventional configuration (1), previously defined and validated,^6,7^ was defined as 40%–60% where <40% is healthy tissue, 40%–60% BZ, and >60% is scar core. Using the same classification scheme, 3 other configurations were applied; threshold 2 (35%–65%), threshold 3 (30%–70%), and threshold 4 (45%–55%). This resulted in 4 LV models per short-axis LGE dataset for each patient. Respective masses of healthy tissue, BZ, and scar core (in grams) for each individual model were computed. In addition, ADAS was used to quantify the number and mass of corridors (also referred to as “channels” or isthmuses), that is, regions of BZ surrounded by scar core, as quantified previously.^7^

A custom-written Python script was used to create 3D volumetric meshes using Meshtool software, by interpolating the information on the tissue characteristics from the exported layers generated from ADAS. For 3 of the patients we were unable to generate volumetric meshes, so these patients were excluded from the interface metric analysis. These meshes were evaluated using Meshtool to quantify 2 metrics, specifically the interface (surface) area (1) between healthy myocardium and hyperenhanced tissue (scar plus BZ), and (2) between BZ and scar core.

### Ventricular ablation

Catheter ablation was performed after discontinuation of all antiarrhythmic drugs, except for amiodarone. ICD therapies were inactivated during the ablation procedure. Substrate mapping was performed using a 3D electroanatomic mapping system (CARTO, Biosense Webster, Irvine, CA) with the PentaRay multielectrode catheter (Biosense Webster). VT induction was attempted using programmed ventricular stimulation at a basic drive cycle length of 500 ms with up to 3 extrastimuli. Ablation was performed at sites with excellent pacemapping correlation, mostly in conjunction with scar homogenization using irrigated catheters (Thermocool SmartTouch DF, Biosense Webster) with irrigation flow rates of 8–17 mL/min at 30–50 W and temperature limit of 45°C. After ablation, noninducibility was tested using programmed ventricular stimulation.

### Statistical analysis

Statistical analysis was performed using IBM SPSS Statistics Version 26. Continuous variables are given as mean ± SD. Independent T test was performed to compare baseline variables between patients with and without recurrence. Repeated measures analysis of variance (ANOVA) was performed to compare intermodel differences in the quantified parameters (BZ mass, scar, total number of corridors and their mass, and the 2 surface metrics) for the 4 PSI configurations. *Post hoc* analysis was performed with Bonferroni correction. Pearson correlation was calculated to study the relationship between the quantified parameters for the 4 PSI configurations and arrhythmia recurrence. Receiver operator characteristic analyses were performed to evaluate the sensitivity and specificity of all parameters. J statistic was calculated to identify the optimal cutoff value with the highest possible sensitivity in parameters with an area under the curve (AUC) of at least >0.7. *P* <.05 was considered significant.

## Results

Twenty-seven patients with a diagnostic LGE-CMR were identified and included in the study. Patient characteristics and ablation strategy are listed in Table 1. LGE-CMR was acquired before the ablation procedure (median 3.9 years; interquartile range 1.22–6.94). The patients had no further infarcts in the interim. Twenty-five of the patients (93%) were male (mean age 72.8 ± 9.5 years). Fifteen patients (56%) had recurrence during the follow-up period of 2.6 ± 1.6 years. Of the total 15 patients with 1 or multiple VT recurrences, only 2 (13%) had a self-limiting tachycardia; the remainder required antitachycardia pacing (n = 6 [40%]) or shocks (n = 7 [47%]) for termination. Additional data regarding device programming and recurrence episodes are provided as Supplemental Table 1. Baseline ejection fraction was significantly lower in the group with recurrence (27.3 ± 8.3) compared to the group without (35.7 ± 4.9) (*P =* .003). LV volumes showed a similar difference between the 2 groups, with significantly higher end-systolic and end-diastolic volumes in patients with recurrence (Table 1). A total of 116 scar maps (4 per patient) were evaluated for subcharacterization of scar parameters and their correlation with arrhythmia recurrence.

### Intermodel differences

**Figure 2:**
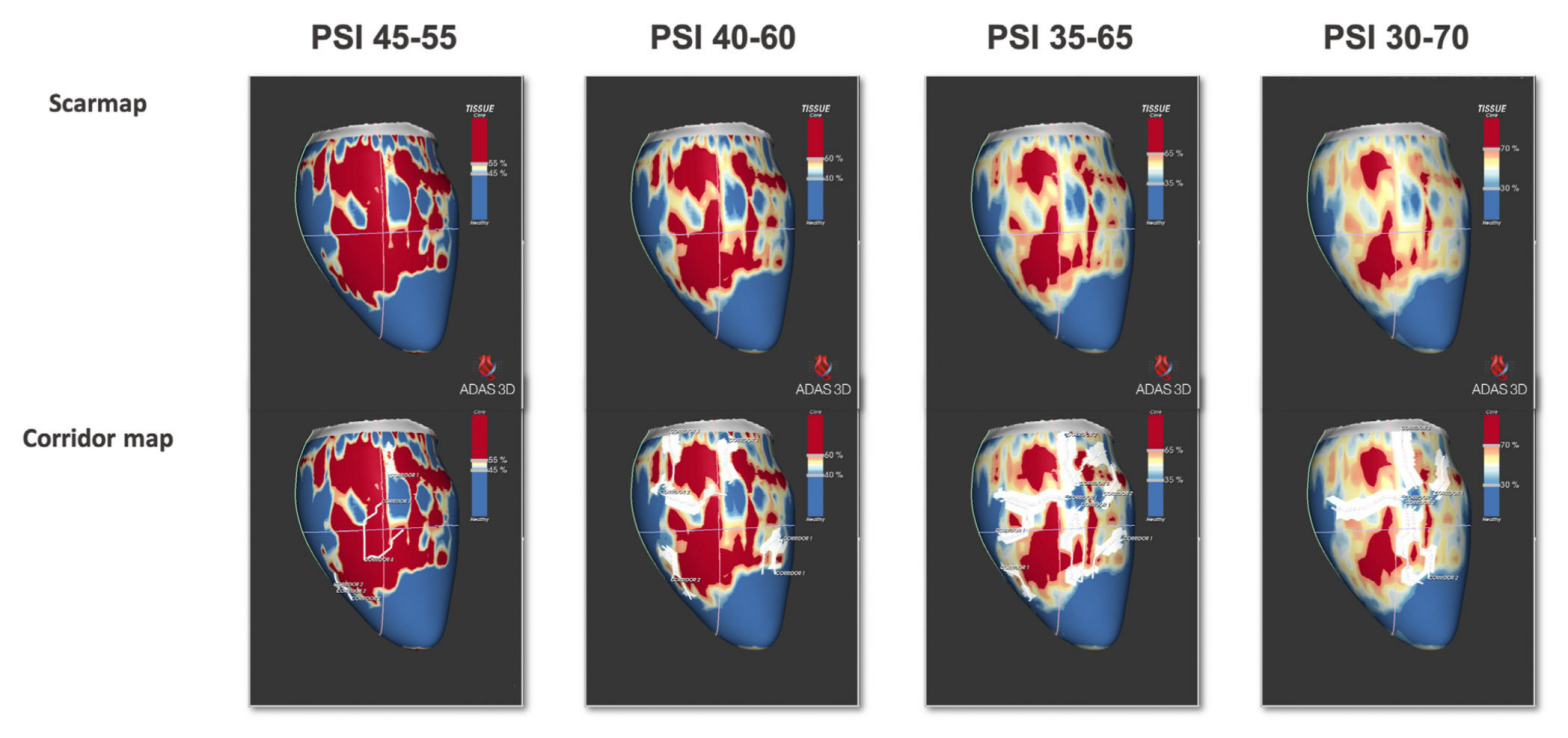
Intermodel differences in scar and corridor map. Scar- and corridor-maps derived from ADAS for the 4 evaluated threshold ranges. *Blue* is healthy myocardium, *red* is scar core, and the remainder is border zone. **Top row**: Increase in border zone mass and decrease in scar with increasing pixel signal intensity (PSI) ranges. The number of corridors. **Bottom row**: Remains comparable between the PSI thresholds.

As expected, BZ mass increased and decreased with increasing PSI ranges (Figure 2). ANOVA demonstrated a significant change in BZ (*P <*.001) and (*P <*.001) between the 4 PSI configurations (Table 2). The number of corridors was comparable among the PSI configurations (*P =* .233). However, a significant change was observed in corridor mass (*P* <.001). Bonferroni adjusted *post hoc* analysis revealed that corridor mass was significantly lower for narrow PSI ranges (*P* <.01).

Repeated measures ANOVA determined that there was a statistically significant difference in the surface area between healthy myocardium and hyperenhanced area for all 4 PSI groups (F = 48.168; *P* <.001). BZ and scar core surface also had a statistically significant difference (F = 16.062; *P* <.001). *Post hoc* analysis with Bonferroni adjustment revealed that the surface area between BZ and scar core decreased significantly between PSI 30–70 and the other groups: PSI 40–60 (10.1 [95% confidence interval (CI); –17.1 to –3.1] cm^2^; *P* = .002); PSI 35–65 (–7.9 [95% CI –12.9 to –2.9] cm^2^; *P* = .001); and PSI 45–55 (–10.7 [95% CI –18.3 to –3.1] cm^2^; *P* = .003). There was no significant difference among the other 3 groups.

### Correlation with arrhythmia recurrence

All 4 groups demonstrated a positive correlation between total enhancement and recurrence, and BZ and recurrence. The strength of the correlation was moderate for all 4 groups, with the strongest relation for the group with the narrowest PSI (45–55). Scar showed a trend toward significance for the PSI 45–55 group, without any significant correlation for the other 3 PSI groups. No significant correlations were observed between the number of corridors or the total corridor mass, and arrhythmia recurrence for the PSI 30–70 and PSI 45–55 groups. The other 2 groups (PSI 40–60 and PSI 35–65) showed a trend toward significance with weak positive correlations of 0.282 and 0.365, respectively.

Surface area metrics showed the best correlation in predicting arrhythmia recurrence with comparable strength and significance between the 4 groups. The strongest relation was observed for PSI 45–55 (r = .458; *P* = .021).

**Figure 3:**
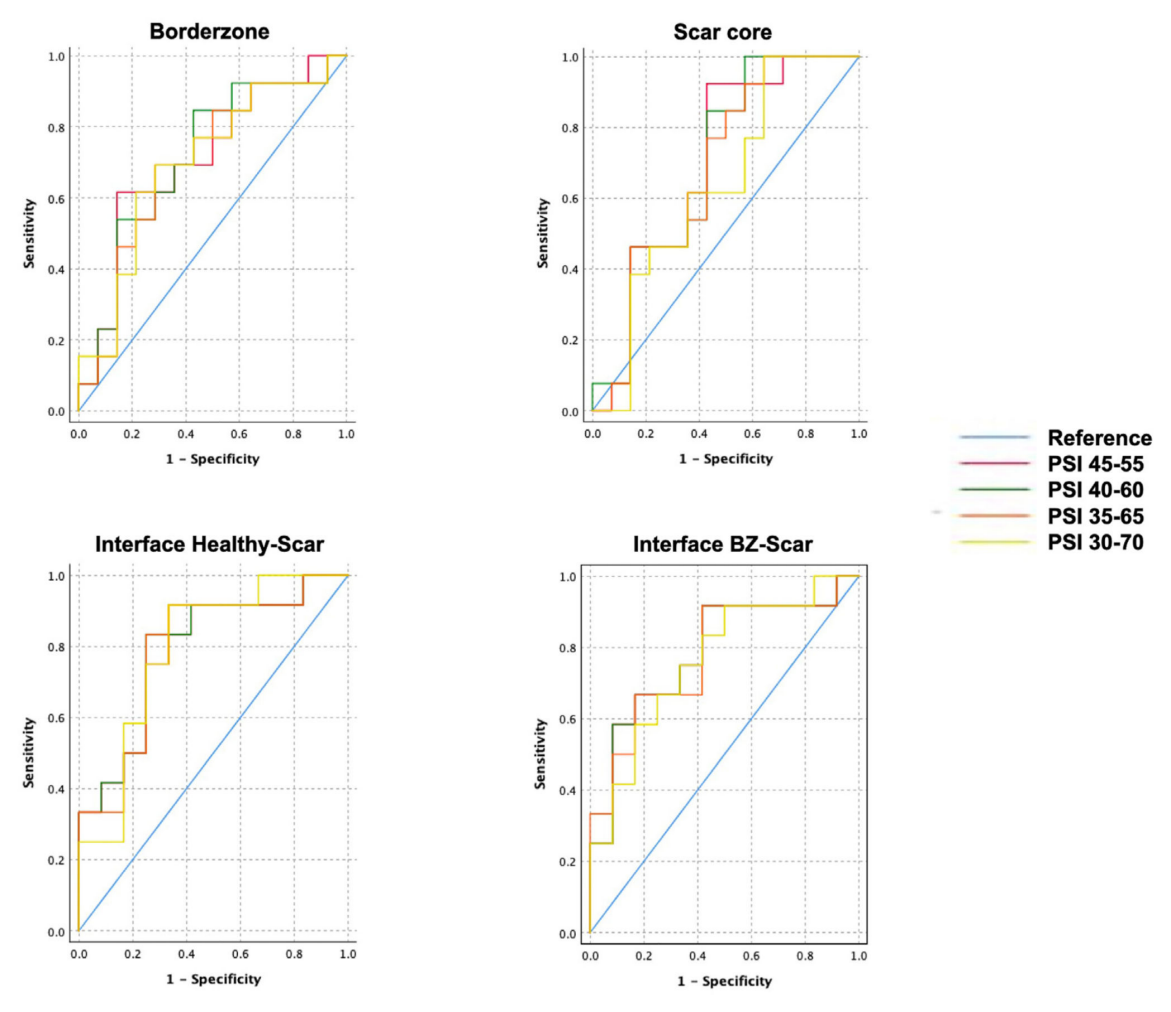
Receiver operator characteristic (ROC) curves relating pixel strength intensity (PSI) thresholds with recurrence. ROC curves depicting an area under the curve between 0.7–0.8 for the hyperenhancement characteristics. PSI group 45–55 demonstrates the best predictive value for recurrence based on the 4 scar metrics: border zone (BZ) mass (**left top**), scar (**right top**), interface between healthy and scar (**left bottom**), and interface between BZ and scar (**right bottom**).

Receiver operator characteristic analysis showed an AUC between 0.7–0.8 for the hyperenhancement characteristics in all 4 models toward predicting recurrence (Figure 3 and Table 3). Scar had the highest J statistic in predicting recurrence, with a cutoff between 2.95–5.72 g depending on the PSI group. PSI 40–60 demonstrated the best predictive value for recurrence based on the cumulative J statistic for total enhancement area, BZ mass, and scar. Surface area metrics had AUC between 0.757–0.821 and demonstrated an overall higher J statistic. The optimal PSI toward predicting recurrence was 35–65, with healthy scar surface cutoff of 74.2 cm^2^ (sensitivity 92.9%; specificity 60%; J = .529) and BZ scar surface cutoff of 110 cm^2^ (sensitivity 50%; specificity 100%; J = .500).

## Discussion

This is the first study to extensively evaluate the influence of different threshold settings in subcharacterizing hyperen-hancement during postprocessing of LGE-CMR and the association of the quantified parameters with arrhythmia recurrence following VT ablation. The main findings are as follows: (1) increasing PSI ranges resulted in increased BZ and decreased scar core without changes in the total number of corridors or their weight; (2) scar metrics quantified using the narrowest PSI range (45–55) had the strongest relation with arrhythmia recurrence following catheter ablation of VT; and (3) surface interface area between healthy and scar, and BZ and scar most strongly predicted arrhythmia recurrence compared with traditional parameters including core mass and BZ mass.

### Image analysis

In general, clinical workup in patients scheduled for VT ablation consists of LGE-CMR to assess the presence and location of arrhythmogenic substrate. As imaging is becoming an integral part in guiding clinical decision-making regarding ablation strategy, it is vital to understand the implications of changing thresholds and the subsequent impact on the generated scar characteristics. The current consensus of postprocessing is based on a study conducted in 2011, performed in a very limited number of patients (n = 10).^6^ All subsequent evidence has simply used the proposed thresholding range without ever performing a cross-validation of this approach.^5,7^ As expected, this study shows that scar characteristics change in a predictable manner with different thresholding settings, that is, increasing BZ mass and decrease in scar with increasing threshold ranges. However, it is important to note that the number of corridors and their overall mass do not seem to be significantly affected. Because corridor analysis is the most relevant aspect of the entire postprocessing strategy, there is no direct clinical relevance of these findings. However, when comparing scar metrics to postablation VT recurrence, it is evident that the narrowest PSI group (45–55) seems to perform the best for identifying patients at risk for recurrence. Therefore, although the different scar thresholding strategies provide comparable and predictable visualization of the scar, PSI 45–55 has the potential to significantly refine patient selection for catheter ablation.

### Interface area vs mass

Multiple studies have investigated the role of BZ and increased arrhythmogenicity in patients with an ischemic cardiomyopathy.^9–11^ BZ mass has been demonstrated to improve prediction of appropriate ICD therapy^9^ as well as ventricular arrhythmias in general for this particular population.^10^ This arrhythmogenic nature of BZ has been described even in patients with LV ejection fraction >35%.^11^ Our study showed comparable results by demonstrating that the extent of BZ has a correlation with arrhythmia recurrence. However, it is important to note that the interface area metrics outperformed the conventional scar quantification with a larger AUC (0.757–0.821) and an overall higher J value. Although interface area is related to BZ mass, it still represents a distinctly different quantity, as demonstrated previously in patients with a nonischemic cardiomyopathy.^12,13^

Based on these findings, it seems possible to accurately estimate VT ablation success from BZ mass and scar interface area. Incorporating these imaging-derived scar characteristics in the workup of VT can significantly improve procedural efficacy. Equally important, this selection strategy could identify patients with a high *a priori* likelihood of postablation recurrence, who might benefit from directly undergoing an alternative ablation strategy such as stereotactic radiation therapy.

### Study limitations

The main limitation of this study is the lack of ground truth (histological analysis) to evaluate which PSI threshold provides the most accurate representation of the myocardium. Although a comparison with histology would identify the best possible threshold settings in each patient, it would not affect the outcome of the current study, which relates imaging-derived scar metrics to arrhythmia recurrence. It is to be expected that the ablation procedure will have a direct impact on the scar characteristics and subsequent (long-term) outcome. In addition, ICD programming can also have an impact on the classification of arrhythmia recurrence and subsequent therapy. However, corridors picked up by ADAS convey scar complexity, which may still be the case postablation. In addition, all patients included in this single-center study consist of ablations performed using a standardized ablation strategy. Considering that this study measured scar complexity and applied a standardized approach for imaging as well as ablation, it is expected that these results truly reflect the impact of imaging characteristics and are not likely to be biased by the ablation strategy. The CMR scanner field strength used in this study was 1.5 T, and postprocessing was performed on a 2D dataset. Although the increased magnet strength provided by a 3-T scanner, in combination with a higher resolution (3D) sequence, could be used to improve input image quality and theoretically could lead to different results, the combination of 1.5 T and 2D is by far the most common method used clinically. Therefore, the analysis performed in this study has significant importance to the current clinical practice. Second, although performed on a relatively small patient cohort with primarily male patients, this work represents the largest study performed to date compared with previous studies evaluating the impact of thresholds on scar characteristics.^6^

## Conclusion

Adapting threshold values have a significant impact on scar, BZ, and surface area quantification, with wider PSI ranges resulting in smaller core and larger BZ mass. Scar metrics quantified using the narrowest PSI range (45–55) have the strongest relation with arrhythmia recurrence, with the interface between healthy and scar and between BZ and scar demonstrating the best predictive value compared with traditional parameters such as core mass and BZ mass. The functional implications of these findings for image-guided ventricular ablation should be investigated in a prospective study.

## Supplementary Material

Appendix

## Figures and Tables

**Table 1:** Baseline and VT ablation characteristics

	Entire cohort (N = 27)	No recurrence (n = 12)	Recurrence (n = 15)	*P* value
Age (y)	72.8 ± 9.2	72.1 ± 9.2	73.4 ± 9.5	NS
Men	25 (93)	11 (92)	14 (93)	
LV ejection fraction (%)	31.1 ± 8.1	35.7 ± 4.9	27.3 ± 8.3	.003
LV EDV (mL)	221.5 ± 48.2	199.6 ± 46.8	239.1 ± 43	.034
LV ESV (mL)	153.3 ± 38.6	129.4 ± 34.9	172.5 ± 30.5	.003
LV mass (g)	123.1 ± 26.7	110.3 ± 20.3	133.3 ± 27.3	.019
Recurrence	15 (56)			
Ablation strategy				
Pacemapping	3 (11)	3 (25)	0	
Substrate based	4 (15)	3 (25)	1 (7)	
Combined approach	20 (74)	6 (50)	14 (93)	
VT inducible during ablation	25 (93)	9 (75)	14 (93)	

Values are given as mean ± SD or n (%) unless otherwise indicated. EDV = end-diastolic volume; ESV = end-systolic volume; LV = left ventricle; NS nonsignificant; VT = ventricular tachycardia.

**Table 2:** Comparison of enhancement characteristics between different thresholding ranges (N = 27)

	PSI 45–55	PSI 40–60	PSI 35–65	PSI 30–70	ANOVA (F value)	*P* value
Total enhancement (g)	24.20 ± 13.82	30.25 ± 17.53	37.39 ± 21.25	44.63 ± 23.69	68.830	<.001
BZ mass (g)	9.08 ± 5.87	18.53 ± 11.79	28.70 ± 17.05	38.64 ± 20.87	90.863	<.001
Scar (g)	15.12 ± 9.14	11.72 ± 7.59	8.68 ± 6.11	6.09 ± 4.54	81.528	<.001
Total corridors	4.81 ± 4.65	6 ± 4.45	5 ± 3.83	4.15 ± 3.17	1.501	.233
Corridor mass (g)	1.38 ± 2.21	4.89 ± 5.22	8.72 ± 8.46	11.73 ± 10.56	30.697	<.001
Surface healthy HE (cm^2^)	97 ± 41	100 ± 43.2	110.6 ± 48.1	123.4 ± 50.7	48.168	<.001
Surface BZ scar (cm^2^)	82.4 ± 38	81.9 ± 38.1	79.7 ± 37.7	71.7 ± 35.1	16.062	<.001

Values are given as mean ± SD unless otherwise indicated. ANOVA = analysis of variance; BZ = border zone; HE = hyperenhancement; PSI = pixel signal intensity.

**Table 3:** ROC analyses relating PSI thresholds with recurrence (N = 27)

		PSI 45–55	PSI 40–60	PSI 35–65	PSI 30–70
Total enhancement	AUC	0.717	0.733	0.700	0.711
	*P* value	.029	.015	.048	.036
	Cutoff	10.16 g	13.85 g	19.45 g	24.38 g
	Sens	100%	93.3%	86.7%	86.7%
	Spec	25%	33.3%	33.3%	33.3%
	J statistic	.250	.266	.200	.200
BZ mass	AUC	0.728	0.733	0.711	0.711
	*P* value	.020	.016	.036	.036
	Cutoff	5.06 g	10.47 g	15.86 g	21.33 g
	Sens	86.7%	86.7%	86.7%	86.7%
	Spec	33.3 %	41.7%	33.3%	33.3%
	J statistic	.200	.284	.200	.200
Scar	AUC	0.733	0.722	0.706	0.656
	*P* value	.019	.029	.045	
	Cutoff	5.72 g	4.22 g	2.95 g	
	Sens	100%	100%	100%	
	Spec	33.3%	41.7%	33.3%	
	J statistic	.333	.417	.333	
Total corridors	AUC	0.619	0.567	0.533	0.417
Corridor mass	AUC	0.619	0.672	0.711	0.689
	*P* value			.035	
	Cutoff			1.63 g	
	Sens			93.3%	
	Spec			33.3%	
	J statistic			.266	
Surface healthy HE (cm^2^)	AUC	0.807	0.814	0.821	0.786
	*P* value	.001	<.001	<.001	.006
	Cutoff	65.7 cm^2^	68 cm^2^	74.2 cm^2^	88 cm^2^
	Sens	92.9%	92.9%	92.9%	92.9%
	Spec	50%	50%	60%	60%
	J statistic	.429	.429	.529	.529
Surface BZ scar (cm^2^)	AUC	0.800	0.793	0.779	0.757
	*P* value	.001	.002	.004	.011
	Cutoff	100 cm^2^	100 cm^2^	110 cm^2^	81.1 cm^2^
	Sens	57.1%	57.1%	50.0%	57.1%
	Spec	100%	100%	100%	90%
	J statistic	.571	.571	.500	.471

AUC = area under the curve; ROC = receiver operating characteristic; Sens = sensitivity; Spec = specificity; other abbreviations as in Table 2.
