# Supplementary material for: Predicting arrhythmia recurrence following catheter ablation for ventricular tachycardia using late gadolinium enhancement magnetic resonance imaging: Implications of varying scar ranges
Source: Heart Rhythm. Author manuscript; Available in PMC 2024 Jul 11. (PMC7616170; doi:10.1016/j.hrthm.2022.05.021)
Supplement: Appendix [file EMS197138-supplement-Appendix.pdf]

Appendix

Supplementary data

Supplementary data associated with this article can be found in the online version at https://doi.org/10.1016/j.hrthm.2022.05.021.
